# Colonic and perianal ulceration exhibiting vacuolar interface dermatitis in the setting of HIV

**DOI:** 10.1002/ccr3.2222

**Published:** 2019-06-22

**Authors:** Joseph Tadros, Cody A. Chastain, Eric Tkaczyk

**Affiliations:** ^1^ University of Cincinnati College of Medicine Cincinnati Ohio; ^2^ Division of Infectious Diseases Vanderbilt University Medical Center Nashville Tennessee; ^3^ Department of Veterans Affairs Tennessee Valley Health System – Dermatology and Research Services Nashville Tennessee; ^4^ Vanderbilt Dermatology Translational Research Clinic, Vanderbilt University Medical Center Nashville Tennessee; ^5^ Department of Biomedical Engineering Vanderbilt University Nashville Tennessee; ^6^ Present address: University of Missouri Columbia MI

**Keywords:** dermatology, HIV/AIDS, immunosuppression, interface dermatitis, perianal and colonic ulceration

## Abstract

We report a case of noninfectious vacuolar interface dermatitis associated with colonic and perianal ulceration in a patient with acquired immunodeficiency syndrome (AIDS), which responded to immunosuppressive treatment. Our findings suggest that interface dermatitis in the setting of AIDS may warrant further gastrointestinal evaluation and may respond to immunosuppression.

## 1 INTRODUCTION

Interface dermatitis is a histopathological term that identifies a primary pathological change along the dermoepidermal junction. It is classically associated with autoimmune conditions, viral exanthems, and eczematous or inflammatory eruptions. Here we report a case of vacuolar interface dermatitis associated with colonic and perianal ulceration in a patient with acquired immunodeficiency syndrome (AIDS), which is unique and not previously reported in the literature. A brief overview of interface dermatitis and potential mechanisms responsible for the classic histologic changes are included.

## 2 CASE PRESENTATION

A 48‐year‐old male with AIDS presented to a community hospital with 3 weeks of increasingly painful oral and perianal lesions after a year of nonadherence with antiretroviral therapy. He was hypotensive and tachycardiac and therefore treated for sepsis with antibiotic therapy. A perianal ulcer and a small ulcer on the anterior midline of the tongue were noted. Intravenous (IV) acyclovir was initiated for presumed herpes simplex virus (HSV). The patient was discharged on oral acyclovir after clinical improvement. His CD4 lymphocyte count was 24 cells/mm^3^. Antiretroviral therapy (ART) consisting of lamivudine, dolutegravir, darunavir, and ritonavir was initiated during the admission.

**Figure 1:**
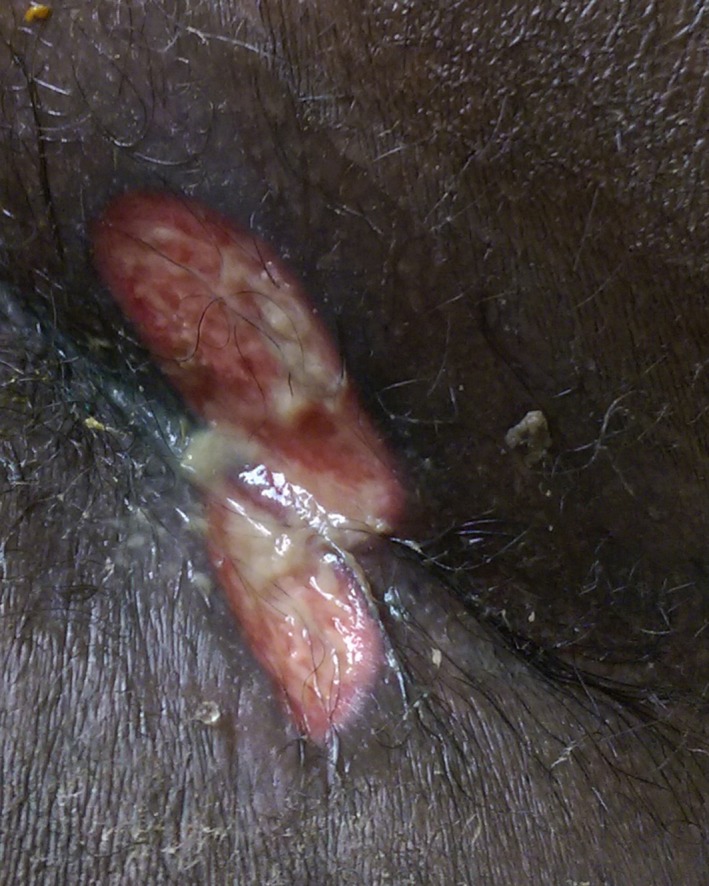
Shallow ulcer with purulent exudate and raised margins in the perianal region

**Figure 2:**
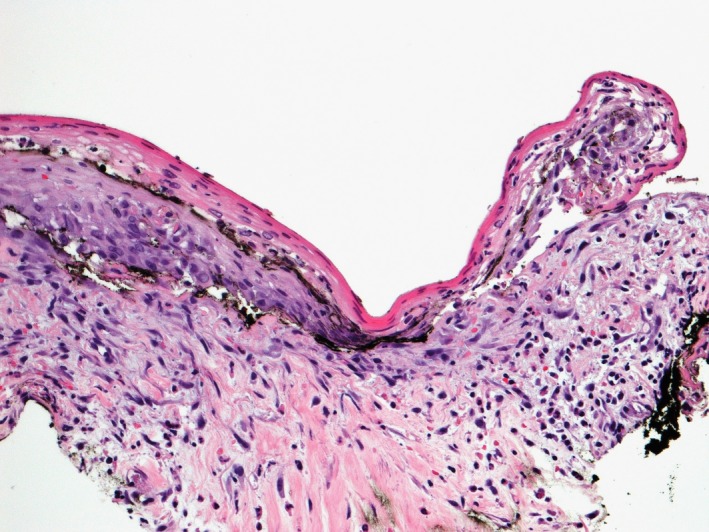
Biopsy specimen demonstrating basal vacuolization, spongiosis, and scattered dyskeratosis within the epidermis consistent with interface dermatitis. A moderate lymphocytic and plasma cell infiltrate at the dermal‐epidermal junction is appreciated (hematoxylin‐eosin, original magnification ×200)

Two days after discharge, he presented to our hospital with worsening rectal pain, fevers, and chills. He was restarted on acyclovir intravenously, and dermatology was consulted. A 4‐cm shallow ulcer on the perianal buttock with fibrinous yellow exudate and raised margins was noted (Figure 1) and biopsied. Histopathology revealed interface dermatitis with basal vacuolization as well as spongiosis and scattered dyskeratotic cells (Figure 2). Periodic acid‐Schiff, cytomegalovirus (CMV), Grocott's methenamine silver, and *Treponema pallidum* infectious stains were negative. Laboratory assessment including rapid plasma reagin, direct fluorescent antibody for HSV and varicella zoster virus, CMV DNA polymerase chain reaction (PCR), and HSV PCR and culture were all negative. A CT abdomen/pelvis performed due to abdominal discomfort did not reveal an etiology of his pain and fever.

Concern for gastrointestinal bleeding prompted an esophagogastroduodenoscopy, which revealed an esophageal ulcer. Colonoscopy revealed punched out ulcers with raised borders and complex bases exposing underlying muscular mucosa at the cecum, ascending colon, and hepatic flexure. Colonic biopsies were negative for CMV, cryptococcus, and histoplasmosis infection; histology revealed ulceration with surrounding inflammation.

His multifocal ulcerative disease was treated with methylprednisolone intravenously followed by an oral prednisone taper with subsequent clinical resolution.

## 3 DISCUSSION

Interface dermatitis has not been previously associated with perianal ulceration in AIDS patients. It has been described in nonmucosal skin of AIDS patients with drug eruptions or seborrheic dermatitis.^1^ Other conditions reported to exhibit interface dermatitis include viral exanthems, phototoxic dermatitis, acute radiation dermatitis, erythema multiforme, erythema multiforme‐like drug eruption, lupus erythematosus, dermatomyositis, and acute graft‐versus‐host reaction.^1^, ^2^, ^3^ Interface dermatitis involves primary pathological changes along the dermoepidermal junction (DEJ) and is classified in two categories based on morphological features: interface dermatitis with (a) lichenoid inflammation or (b) vacuolar change.^3^, ^4^ The latter category is characterized by individual cell necrosis of primarily basal keratinocytes and infiltration of inflammatory cells along the DEJ.^5^ It is hypothesized that the wide range of clinical conditions associated with this histopathology is due to a common mechanism stemming from cellular cytotoxicity of keratinocytes via CD8 T cell—mediated destruction, antibody‐dependent cellular cytotoxicity, or autoantibodies targeting cellular components of the basement membrane.^4^, ^6^ Additionally, colonic ulceration is typically observed in the context of opportunistic infections, including CMV reactivation, bacterial colitis, HIV‐related cryptococcosis, or histoplasmosis—none of which were detected in this case.^7^

Alternative diagnoses, including possible manifestation of immune reconstitution inflammatory syndrome (IRIS) or erosive lichen planus, should be considered given there was no infectious cause identified to explain the HIV‐associated perianal ulceration. IRIS is unlikely given our patient's systemic symptoms and painful perianal ulceration began well before ART initiation.^8^ Erosive lichen planus (LP) is a possibility, but would not be expected to associate with colonic ulcers. A lack of a lichenoid infiltrate on biopsy also makes this diagnosis unlikely. Lastly, there have only been a few rare case reports of LP seen in patients with HIV, and no recent increase in prevalence has been identified.^9^, ^10^, ^11^

## 4 CONCLUSIONS

While it is well known that the gastrointestinal tract harbors abundant lymphoid tissue and subepithelial Langerhans cells that facilitate HIV‐1 colonization, the mechanisms of direct viral damage to mucosal and epithelial keratinocytes are not well understood.^12^ Our case suggests that HIV may directly cause mucosal and skin pathology via alternative, autoimmune mechanisms. If all possible infectious etiologies of perianal ulceration are excluded, the finding of interface dermatitis in the setting of AIDS may warrant further gastrointestinal evaluation and initiation of therapeutic immunosuppression.

## CONFLICT OF INTEREST

None declared.

## AUTHOR CONTRIBUTIONS

JT: Involved in literature search, preparing manuscript for submission, organizing and gathering data, drafting and revising manuscript. CAC: Involved in initial workup of patient, final approval for publication, data acquisition, drafting and revising manuscript. ET (Corresponding Author): Involved in initial workup of patient, organizing and gathering data, final approval for publication, hospital course and clinical/histopathology photo retrieval, drafting and revising manuscript. Writing and revising the manuscript as a contribution also by both CAC and ET.
